# Social incentives as nudges for agricultural knowledge diffusion and willingness to pay for certified seeds: Experimental evidence from Uganda

**DOI:** 10.1016/j.foodpol.2023.102506

**Published:** 2023-10

**Authors:** Julius Okello, Kelvin Mashisia Shikuku, Carl Johan Lagerkvist, Jens Rommel, Wellington Jogo, Sylvester Ojwang, Sam Namanda, James Elungat

**Affiliations:** a International Potato Center, Kampala, Uganda; b International Livestock Research Institute, Nairobi, Kenya; c Department of Economics, Swedish University of Agricultural Sciences, Uppsala, Sweden; d International Potato Center, Lilongwe, Malawi; e International Potato Center, Nairobi, Kenya; f Ministry of Agriculture, Animal Husbandry and Fisheries, Katakwi District Local Government, Uganda

**Keywords:** Social incentives, Goal setting, Nudge, Willingness to pay, Adoptio`n, Uganda

## Abstract

A transition from low-input subsistence farming in Sub-Saharan Africa will require the use of yield-increasing agricultural technologies. However, in developing countries, most farmers continue to rely heavily on pest-infested and disease-infected recycled seed from own or local sources leading to low yields. This study used a field experiment to examine the effect of a social incentive combined with goal setting on the diffusion of agricultural knowledge and uptake of quality certified seed by farmers. We relaxed the seed access and information/knowledge constraints by introducing improved varieties of sweetpotato in the study villages and providing training to carefully selected progressive farmers who were then linked to co-villagers. We find that social incentives combined with goal setting reduced the likelihood of the trained progressive farmers reaching out to co-villagers to share information and discuss farming. Further, social incentive combined with goal setting had no significant effect on knowledge and experimentation by progressive farmers, and on willingness to pay for improved seed – as elicited through auctions, our proxy for experimentation, by co-villagers. These findings suggest that the combination of goal setting and public recognition acted to crowd-out diffusion effort. We conclude that social incentive combined with goal setting by established progressive farmers already enjoying a certain degree of public recognition is not sufficient to induce effort in learning and experimentation with agricultural innovations. These results have implications for design of policy and extension services to promote adoption of agricultural technologies with proven food and nutrition security benefits in developing countries.

## Highlights

• We examine effect of social incentives on social learning and quality seed technology adoption.
• A field experiment involved training of model farmers, goal setting, and provision of public recognition.
• Model farmers were less likely to contact co-villagers when incentivized with public recognition.
• Farmers’ knowledge and uptake of quality seeds were unaffected by social incentives.
• Findings suggest social incentive crowded-out diffusion effort.

## 1 Introduction

Improving the welfare of millions of farming households in the developing countries ultimately requires a shift from subsistence, low-input agriculture, to commercial agriculture that relies on productivity-increasing technologies. In developing countries, access to and use of quality certified seed, a key input into the production process, remains a major challenge (Bold et al., 2017, McGuire and Sperling, 2016, Mwangi et al., 2020, Sperling et al., 2020). In Sub-Saharan Africa (SSA), for instance, most farmers rely on poor quality own saved/recycled seed or seed obtained from local sources (Jaleta et al., 2020, McGuire and Sperling, 2016, Schulte-Geldermann et al., 2012). The low variety turnover due to limited use of improved varieties (IVs) with yield advantages and greater adaptability to abiotic and biotic stresses than non-improved varieties has been associated with high yield gaps in developing countries (Gildemacher et al., 2009). Unlike the green revolution rice varieties of the 1960s and 1970s that met high adoption rates, recent IVs of staple crops have encountered much lower adoption rates. This is particularly the case for IVs of root and tuber crops, a group of crops (namely cassava, potato, yams and sweetpotato) seen as very important for the low-rainfall marginal areas of SSA with highly variable climate. McEwan et al. (2021), for instance, find an adoption ceiling of 40% for IVs of root and tuber crops. For these crops, only about 6% of farmers use improved varieties (Thiele et al., 2021).

Recycled and locally sourced seed are often heavily infested with pests and diseases (Mbewe et al., 2021). Planting diseased low-quality seed can result in high yield penalties and welfare-reducing effects in developing-country agriculture (Mann and Warner, 2017, Okello et al., 2017, Wossen et al., 2020). This can seriously compromise policy and development efforts aimed at achieving food and nutrition security. One of the major contributors to recycling of non-hybrid crop varieties is the large number of landraces (also known as farmer varieties) maintained by farmers (Smale et al., 2001, Asrat et al., 2010). In sweetpotato, for instance, Zawedde et al. (2014) report that farmers, on average, maintain four varieties per plot per season, with the majority of them being landraces. Asrat et al. (2010) find that environmental adaptability and yield stability are major drivers of farmers’ continued demand for local varieties, not least because of a better fit to local conditions. Recent efforts aimed at improving on-farm productivity, increasing incomes, and reducing food insecurity, therefore, target the replacement of farmer varieties with new (genetically improved) varieties. At the same time, project level efforts in many developing countries focus on “cleaning” the existing IVs and re-introducing them in the communities. That is, popular IVs that have been grown in the farming system for several years, and which have accumulated diseases or pests are returned to the lab, the diseases and pests are screened off, and the clean varieties are re-disseminated to farmers.

Farmers’ decision to adopt IVs is often constrained by unavailability, inaccessibility, lack of awareness about the advantages of growing quality seed especially with regard to plant health, and limited knowledge about the sources of improved varieties and how to maintain quality seed on-farm for greater yields. In this study, we addressed the seed availability and informational constraints. Working with the local government, we conducted an experiment that carefully selected progressive farmers, henceforth referred to as disseminating farmers (DFs), in Uganda and trained them in the production and marketing of improved sweetpotato varieties. In our context, progressive farmers typically work with public frontline extension staff to promote new agricultural technologies and farming practices in their respective villages. They readily share knowledge with co-villagers, are easily accessible, literate, and live in the villages they represent. We then created information exchange links by matching each trained DF with 11 other farmers, randomly selected from their respective villages. A random subsample of the trained DFs (the treatment group) was asked to set a personal goal indicating their motivation for reaching out to their co-villagers with the knowledge acquired. In addition, this random subsample was promised a social incentive in the form of public recognition as a reward for their effort in helping other farmers learn about the improved varieties. Improved sweetpotato planting material (also known as vines) were then sourced from a certified seed producer and made available in all study villages for purchase at the start of the rains. The objective was to examine the effect of public social recognition combined with goal setting on the diffusion of agricultural knowledge and smallholder farmers’ uptake of quality certified seeds.

The literature on the role of social learning in the diffusion of agricultural innovations emphasizes the importance of incentives in motivating effort (Kondylis et al., 2017, BenYishay and Mobarak, 2019, Beaman et al., 2021, Takahashi et al., 2020, Balew et al., 2022). It suggests that incentivization of carefully selected farmers as entry points for the dissemination of agricultural innovations can spur stronger diffusion of knowledge and stimulate uptake of improved agricultural technologies among farmers than without incentives (BenYishay and Mobarak, 2019, Balew et al., 2022). The effectiveness of such incentives depends on, among others, the nature of the technology (i.e., perceived benefits), its riskiness (Munshi, 2004), social distance between the communicator and other farmers (Feder and Savastano, 2006, Santos and Barrett, 2010, Shikuku, 2019, Kabirigi et al., 2022), and environmental conditions (Munshi, 2004, Magnan et al., 2015). The focus has largely been on material rewards (see for example, BenYishay and Mobarak, 2019, Beaman et al., 2021). These studies document robust evidence that material incentives increase both social learning and experimentation with improved technologies. However, limited attention has been paid to the role of social incentives in the diffusion of agricultural innovations. Notable exceptions include Shikuku et al. (2019) and Balew et al. (2022). Both studies found that public recognition of effort increased diffusion of agricultural innovations. Balew et al. (2022) further showed that the effect of social incentives was larger when framed as a loss. Still, empirical evidence on the role of social incentives in the diffusion of agricultural innovations is narrow and insights from studies outside agriculture indicate that public recognition can crowd-out intrinsic motivation and reduce performance (Savary and Goldsmith, 2020, Wu and Jin, 2020). For example, social incentives can crowd-out intrinsic motivation via the over-justification effect, a phenomenon that arises from losing the benevolence spirit after feeling targeted by change agents/communicators (McRaney, 2011).

Our study complements a narrow but rapidly growing strand of research testing approaches for motivating workers to expend costly effort in implementing prosocial tasks associated with the diffusion of agricultural innovations (BenYishay and Mobarak, 2019, Shikuku et al., 2019, Balew et al., 2022). These studies have shown that material rewards and social incentives increase the diffusion of agricultural innovations. Our study provides the first evidence of the effect of combining goal setting and social incentives on the diffusion of agricultural innovations. Contrary to previous studies, we find a negative relationship between goal setting combined with public recognition and the diffusion effort suggesting possible crowding out effects. Further, we find that the crowding-out effect was stronger for certain improved varieties but not others indicating that the treatment effect of the social incentive is heterogenous, corroborating the findings of Magnan et al. (2015) and Kondylis et al. (2017).

Our study is closest to Shikuku et al., 2019, Shikuku, 2019, and Shikuku and Melesse (2020), because they are all implemented with farmers in Uganda and provide social recognition to DFs as an incentive for the diffusion of agricultural knowledge. Shikuku et al. (2019) and Shikuku and Melesse (2020) study innovations in drought-resistant maize, with training treatments distinguishing material and reputational rewards. Shikuku (2019), using quasi-experimental techniques, studies the impact of social distance on knowledge diffusion. However, in addition to focusing on a different crop (namely, sweetpotato), there are important unique features of our study design making it complementary to the earlier studies. First, the DFs in our study are progressive farmers who have worked with the government extension officers before and therefore may already enjoy some status in the community. This selection criterion is different from that used in previous studies, which instead targeted randomly selected DFs. Second, the social recognition incentive in the previous studies included a reward for the co-villagers because of the DF’s effort. In our study, co-villagers did not receive a reward. Instead, we combine the social recognition with goal setting as a commitment device. Finally, our study deliberately matches each DF to a group of identified co-villagers. Therefore, DFs can choose to focus only on the assigned co-villagers. Previous studies did not disclose the names of the co-villagers with whom the DF was matched. These differences in design features might explain why we observe different results from those of previous studies. For example, while previous studies find strong positive effects of social recognition on DF’s diffusion effort, we document a negative relationship of the social recognition combined with goal setting.

## 2 Study context and local sweetpotato varieties

This study was conducted among sweetpotato farmers randomly selected from Katakwi district in Uganda. Katakwi is one of the districts of the Teso sub-region which is the leading producer of sweetpotato and where the crop is the second most important food staple after cassava (UBOS, 2019). It is estimated that at least 90% of the households, including those in urban settlements, grow sweetpotato. Sweetpotato is mainly consumed in the district in boiled form, but it is conserved dry for off-season use as dried chips and also processed into flakes, popularly known as *amukeke* and *inginyo*, respectively (Echodu et al. 2019). More than 50% of sweetpotato farmers participate in a wide range of markets including local (i.e., farm gate and roadside) and distant urban markets (Engoru et al. 2005). Farmers grow a wide range of varieties in the district, as in the rest of Uganda (Okello et al., 2022).

A farm household maintains, on average, four varieties of sweetpotato in a season (Zawedde et al., 2014). Farmers mainly use seed/vines from their own sources which are typically recycled for several years and, hence, heavily infected with diseases and pests. Consequently, yields are quite low – averaging 4.3 tons/hectare compared to an achievable 18 tons/hectare under farmers’ conditions and 30 tons/hectare in experimental stations (Naylor et al., 2004, Ngailo et al., 2019).

In this study, we wanted to resolve the constraint of unavailable good quality sweetpotato seed in the district by introducing four virus-free sweetpotato varieties that had been inspected/tested and verified as clean. The varieties (later known as products) were Ejumula, Tanzania, Narospot 1 and Naspot 13. *Ejumula* is a local landrace native to Katakwi district, hence, it is widely grown because of its adaptation to the local environment. It has cream skin and orange flesh, because it is rich in beta carotene, a precursor for vitamin A. However, seed obtained from own or other farmers’ fields are infected with devastating viruses (especially sweetpotato virus diseases – SPVD) and pests (the sweetpotato weevil) and therefore low-yielding. The introduced seed, though of the same genetic identity, was superior to the local seed due to absence of SPVD and weevils. *Tanzania* variety is also very popular and widely grown in Katakwi district and the Teso sub-region. It has a yellow flesh color and a cream skin. The introduced variety was also free from SPVD and weevils, and hence higher yielding than locally available seed from farmer’s own sources or other farmers’ fields.

*Narospot 1* and *Naspot 13* were new introductions in the district. They had not been previously promoted in Katakwi district. Narospot 1 has white flesh and red skin. It was also tested and validated to be free from SPVD and weevils. Lastly, Naspot 13 has orange flesh and cream skin. It was also a new introduction to Katakwi. As with others, the seed used in the auction had been tested and verified as pest and virus-free.

The uptake of new varieties is a process that requires knowledge of its advantage (including yield, income, and nutrition) over the existing varieties. The weakness of the public extension programs is commonplace in developing countries, including Uganda (Ragasa et al., 2016, Namyenya et al., 2022). The Parish development model of the government recognizes the importance of working with progressive farmers to strengthen the extension system. Hence, we used trained champion farmers (namely the DFs) to disseminate information about these improved sweetpotato varieties as discussed in the section below.

Katakwi is one of the districts in Uganda that is a confluence of climatic and sociocultural fragility that influences behavioral outcomes. A large part of the district borders the Karamoja region and inherits two key contributors to fragility, namely, inadequate and erratic rains, and conflicts resulting from frequent cattle raids. Roy et al. (2022) argue that climate fragility exacerbates conflicts and undermines prosocial behavior. Crudeli et al. (2022) argue that social norms stimulate adoption of innovation. However, living in conditions of prolonged hardships and suffering can also result in the phenomenon known in social psychology as *collapse of compassion* where the propensity to help others – i.e., to be prosocial – diminishes with an increased number of people needing support (Cameron and Payne, 2011). Lim and DeSteno (2016) and Vollhardt and Staub (2011) on the other hand argue that adverse life experiences generate heterogenous life outcomes that either enhance or diminish prosocial behavior. These climatic and socio-cultural and behavioral factors influence technology adoption (Crudeli et al., 2022).

## 3 Methodology

### 3.1 Theoretical approach: Social learning, incentives, and prosocial behavior

In order to develop a framework that would generate empirical predictions for our experiment, the study combined insights from the standard target input model (Bardhan and Udry, 1999, Bandiera and Rasul, 2006), a model of communication proposed by BenYishay and Mobarak (2019), and a model of prosocial behaviour (Bénabou and Tirole, 2006).

Farmers are assumed to currently operate using a sweetpotato variety whose payoffs they know well, but with which they are more vulnerable to pests and diseases and with low nutrition benefits. We assume that there exists an improved variety, but its suitability to farmers’ agricultural activities is unknown. Specifically, farmers do not know the target inputs required to implement the improved variety and the associated nutrition benefits. Farmer i has initial beliefs about the improved variety. Based on these prior beliefs the farmer maximizes expected payoffs by implementing what he or she expects to be the target. Expected payoffs from the improved variety decline the farther away the farmer is from the target. The farmer will, therefore, seek to learn in order to correctly estimate the target, hence maximizing payoffs with the improved variety.

Suppose that there exists an informed farmer j who knows the target, denoted $x^{*}$. To communicate this information, the informed farmer sends a signal $s_{\mathrm{ji}}$ incurring a cost c that is increasing in the precision of the message (γ). Following BenYishay and Mobarak (2019), the signal can formally be represented as $s_{\mathrm{ji}}=x^{*}+\left[\frac{\left|j-i\right|}{\gamma }\right]$ where $\left|j-i\right|$ signifies proximity between farmers i and j in terms of similarity in agricultural conditions so that the message received from the communicator is relevant to agricultural decisions of the receiver. Upon receiving the signal, farmer i updates his or her beliefs about $x^{*}$. Accordingly, expected payoffs from learning about the improved variety increase with proximity to the communicator and the precision with which the signal is sent (BenYishay and Mobarak, 2019).

Our *first prediction* is that providing training to DFs will expand their knowledge, subsequently increasing the likelihood of experimenting with the improved variety (Shikuku et al. 2019). Furthermore, a trained DF will likely appear a trustworthy source of knowledge to other farmers (Buck and Alwang, 2011). In addition to increasing signal precision, training might intrinsically motivate a DF to share information with other farmers (Shikuku and Melesse, 2020). Transmitting the signal, however, involves a costly effort. A DF’s engagement in a costly activity to train other farmers is a prosocial task; the task creates benefits enjoyed by those other than the DF (Ashraf et al., 2014). Intrinsic and reputation motivation can influence DFs’ performance in the prosocial task (Benabou and Tirole, 2006). An individual DF chooses a level of effort e involving a cost C(e) to produce an output z and yielding a reward rz. Following the model of prosocial behaviour (Bénabou and Tirole, 2006), the DF solves the following problem:

(1) $$max\left\{\left(v_{z}+v_{r}r\right)z-C\left(z\right)+\mu_{z}E\left(v_{z}|z,r\right)-\mu_{r}E\left(v_{r}|z,r\right)\right\}$$

where $v\equiv \left(v_{z},v_{r}\right)$ represents each DF’s preference type drawn independently from a continuous distribution with density $f\left(v\right)$ and mean $\left({\underset{\_}{v}}_{z},{\underset{\_}{v}}_{r}\right)$; $v_{z}$ denotes the intrinsic valuation of the DF for contributing to the social good, and $v_{r}$ his or her intrinsic valuation for material reward (Bénabou and Tirole, 2006); $\mu \equiv \left(\mu_{z},\mu_{r}\right)$ represents the DF’s reputational concerns with $\mu_{z}=m\delta_{z}$ and $\mu_{r}=m\delta_{r}$; m>0 can be interpreted as a measure of the visibility of the DF’s actions, that is, the probability that the actions will be observed by others or the number of people who will hear about his or her work. The weight attached to social approval and material reward are $\delta_{z}\geq 0$ and $\delta_{r}\geq 0$ respectively.

Eq. (1) shows that engaging in a prosocial task has a direct payoff $\left(v_{z}+v_{r}r\right)z-C\left(z\right)$ and a reputational payoff $\mu_{z}E\left(v_{z}|z,r\right)-\mu_{r}E\left(v_{r}|z,r\right)$. The signs of $\mu_{z}$ and $\mu_{r}$ reflect the idea that people would like to appear as prosocial and not greedy (Benabou and Tirole, 2006).

It has long been thought that observability and public recognition for prosocial behavior increases willingness to act prosocially (see Andersson et al., 2020 for a review of the literature). Several reasons motivate these thoughts. First, self-signaling theory (Bénabou and Tirole, 2006, Bodner and Prelec, 2003) suggests that, in certain situations, people choose options to signal information to themselves about their own characteristics, independent from the desire for the actual outcome (Dubé et al., 2017, Savary et al., 2015). Therefore, one reason that DFs may choose to expend costly effort to engage in a prosocial behavior is to signal to themselves that they are compassionate and altruistic.

Second, social signaling motive suggests that prosocial behavior can be driven by the desire to communicate positive information about the self to neighbors. Studies have shown that people believe engaging in prosocial behavior in front of others will improve their personal reputation (Lacetera and Macis, 2010). The promise of social benefits can motivate people to engage in prosocial behaviors (Grant and Gino, 2010), especially when the public recognition is by members of a valued in-group such as village chiefs. Thus, research on self- and social-signaling motivations has demonstrated that both can positively affect the tendency to engage in prosocial behavior. This is to say that, if a DF wants to engage in a prosocial behavior in order to signal positive information to the self, the likelihood of the DF engaging in the behavior should only increase if the behavior will be observed, because then the same behavior can also signal positive information to others.

Although nudges have been shown to effectively promote prosocial behavior in many situations, recent work suggests that public recognition undermines the intrinsic motivations for altruistic acts (Savary and Goldsmith, 2020, Wu and Jin, 2020). A nascent but growing literature shows that for an action to be seen as altruistic and not “tainted”, it must be perceived as benefiting others without the giver receiving anything in return (Barasch et al., 2016). This means that public recognition can be a self-benefit. The implication is that receiving any form of public recognition can cast doubt on one’s altruistic motivations for contributing to a prosocial task. Consequently, people may be less able to conclude that their costly effort in a prosocial task is motivated by compassionate and altruistic motives, undermining the self-signaling utility from engaging in the task, and reducing the likelihood of conducting the task. This perspective is consistent with the notion that extrinsic incentives can “crowd-out” the motivation to engage in intrinsically motivated behaviors (Bénabou and Tirole, 2006). Similarly, public recognition may also be considered a form of extrinsic personal benefit, which could crowd-out intrinsically motivated actions (Bénabou and Tirole, 2006). In addition, asking people to set a goal as a commitment device can be perceived as “bad news” if it sends a signal that there is lack of trust in the intrinsic motivation of the DF (Bénabou and Tirole, 2006). In such a case, $v_{z}$ in Eq. (1) declines and negatively affects performance.

Furthermore, people may react with a psychological reactance in situations where they feel that they are being manipulated into making certain choices (Gråd et al., 2021). Although nudges, in theory, should preserve the freedom of choice, they can still amount to some level of pressure or result in a feeling that one should behave in a certain way. Because people derive additional utility from behavior that enhances their reputation (Bénabou and Tirole, 2006, Ellingsen and Johannesson, 2008, Frey and Oberholzer-Gee, 1997), nudges could plausibly also crowd out prosocial behavior simply because nudged prosocial behavior generates less utility than non-nudged prosocial behavior in terms of reputational benefits. An altruistic act may be viewed as less altruistic when being nudged, making it less attractive (Wu and Jin, 2020).

In addition to crowding out actual prosocial behavior, nudges may also crowd out warm glow, i.e., people deriving emotional satisfaction from giving, valuing the effort exerted for others rather than the benefit others receive from that effort (Andreoni, 1989, Andreoni, 1990). Since warm glow helps people to maintain a self-image of being moral and fair-minded, an essential factor is how people perceive their own actions and the motivations behind them. Thus, if someone feels pressured or tricked into an action, the prosocial act might be less rewarding in terms of experienced warm glow. It could also induce other unintended negative reactions such as avoidance behavior (Andreoni et al., 2017, Damgaard and Gravert, 2018). Nudges may make an altruistic act more likely to be experienced as ‘giving in’ rather than spontaneous ‘giving’ (see Cain et al., 2014). The effect of public recognition on the effort of DFs to engage in a prosocial costly task to train their co-villagers is therefore an empirical question.

### 3.2 Sampling

**Fig. 1:**
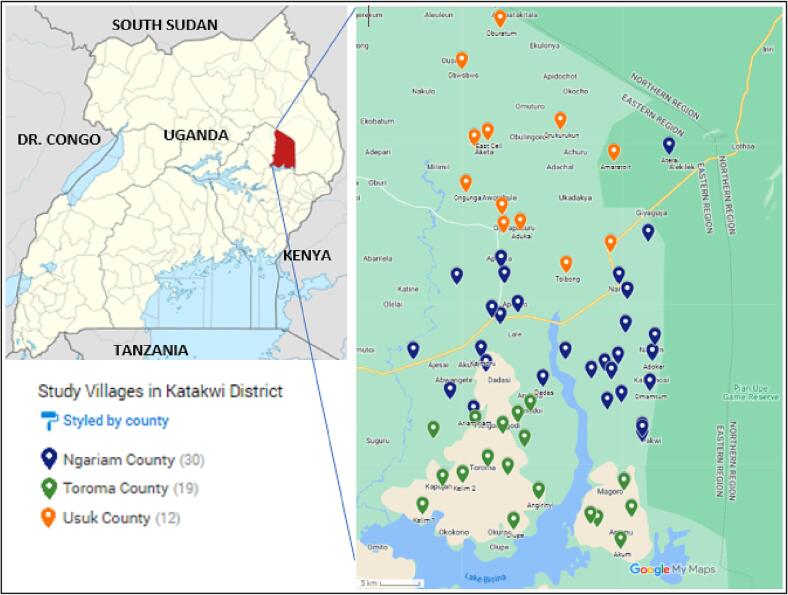
The parishes/villages selected for the auction events.

The study targeted all the three counties and 20 sub-counties in Katakwi district. Within the sub-counties, we randomly selected 61 parishes (there are 131 parishes in total in Katakwi district) from the 20 sub-counties proportionate to size of the sub-county (Fig. 1 shows the distribution of the parishes selected). Next, we randomly selected one village from each parish. With the help of local administrative staff and agricultural offices, we generated a list of all the households that planted sweetpotato in the one year prior to the study in each selected village. This list constituted the sampling frame from which we randomly sampled 11 households in each village to participate in the study. Within the household, the member who planted sweetpotato and was responsible for decision making was then selected.

We also recruited one model farmer in each village to act as the DF. The DFs were recruited by local government staff as described below, using a predetermined criterion developed jointly with the project team. The criteria used for the selection of the DF was: (i) good reputation/standing in the community, ii) perceived as a role model or lead farmer by the local agricultural and administrative office, (iii) ability to communicate and willingness to share information with other farmers and to read and write, iv) resides in the village and, v) planted sweetpotato in the year preceding the study. The total sample size was, therefore, 732 farmers comprising 61 DFs and 671 co-villagers. Some of the DFs had worked with the government and non-governmental organizations in implementing agricultural programs.

### 3.3 Treatment assignment and intervention roll-out

The 61 selected DFs were randomly placed into two groups, the treatment group (T1, n = 30) and control group (T0, n = 31). Both groups were offered a half-day in-room training on sweetpotato vine production broadly covering agronomic, marketing, and utilization aspects. Training for the two treatment groups was conducted on different but consecutive days to avoid contamination. The training was delivered by a senior sweetpotato agronomist. The in-room training was followed by a field visit to a sweetpotato seed multiplier in which the concepts and information learned earlier were reinforced and further clarified through practical demonstration. The training was organized around the following topics: sources of quality sweetpotato seed; yield advantage associated with planting quality seed of improved sweetpotato varieties; how to plant vines/seed (i.e., spacing) to optimize yield; how to monitor vine growth and health during the season; moisture and nutrient management, harvesting and postharvest management; diagnosis and control of pests and diseases especially weevils and sweetpotato virus diseases (SPVD); the health benefits of the biofortified orange-fleshed sweetpotato (OFSP); and the costs and expected payoffs of planting improved variety compared with the local varieties.

At the end of the training, DFs in the treatment group were asked to set goals regarding how they planned to accomplish the task of passing the knowledge acquired to co-villagers. Specifically, each trained DF in T1 group was asked to think about and formulate a “mission statement” explaining briefly how she or he aimed to reach out to other farmers to share new knowledge learned. They were then promised a reward in the form of public recognition if they reached many co-villagers and helped improve their knowledge above a pre-determined threshold. Specifically, T1 DFs were informed that during the auction (to be organized at the start of the rains), the other farmers they had been matched with would be asked a few questions about sweetpotato farming, and that if the average knowledge of the farmers’ in their village met or exceeded a pre-determined threshold (i.e., farmers getting at least 50% of the knowledge questions correct), they would be publicly acknowledged and their photo, name, and “mission statement” would be displayed in the village administrator/chief’s office for two cropping seasons with a message recognizing them as champions of promoting improved sweetpotato varieties in their villages. The content of the knowledge exam was not revealed to the T1 DFs. DFs in the control group received only the in-room and on-field training. They were not promised any reward and did not participate in the goal setting exercise.^1^

We consider the information-only arm as the control group. The decision to use the information-only group as a comparison group instead of including a third arm (pure control) was made to increase statistical power, especially because the randomization was done at the village level and not the individual level. In addition, we wanted to avoid a situation where auctions/surveys are performed without the involvement of DFs for ethical reasons, as a pure control could have undermined trust in DFs operating in the village. It would also be unethical to provide training to one set of DFs (T1) while excluding others (T0) because of the widespread problem of low sweetpotato yields in the whole study district. Our experiment, therefore, provided training to all DFs, but varied the social incentive received to disseminate knowledge. Specifically, our study was structured as follows: (a) all DFs receive the same in-room and on-field training (i.e., information provision/dissemination), and (b) DFs in T1 are then asked to think about and formulate a mission statement related to their role as facilitator of knowledge sharing (this is goal-priming) while knowing about a social recognition reward to be attainable. Thus, DFs in T0 were not goal-primed while their counterparts in T1 were goal-primed. Our approach is therefore based on the seven principles of goal activation and goal priming (Förster et al., 2007). Based on the approach outlined in Förster et al. (2005), the goal-priming and the public recognition is to be understood as “one entity” in which goal setting is primed with the reward that the social recognition brings to the DF. Hence, from a psychological perspective, it is not meaningful to consider goal-setting and social recognition as separate aspects of the study design (albeit from a pure experimentalist/internal validity perspective this may be desirable indeed). Nonetheless, it is worth acknowledging that alternative specifications of the reward could have induced other behavioral responses by the DFs in T1, which indeed could be different from the one that we have observed. We therefore decided to confound these aspects, as they are conceptually inseparable in the psychological literature informing our treatments. We, however, conducted several analysis to try to unpack some of these behavioral aspects. For instance, we assessed the relationship between other farmers’ (i.e., co-villagers') willingness to pay and being contacted by a DF (see Appendix Table A2). We assume that not being contacted by a DF means that the farmer was not exposed to the knowledge that the DF acquired. This is a plausible assumption given the short period of time between DFs training and the willingness to pay experiments. We further assessed the relationship between knowledge scores of other farmers and whether or not they had contact with DFs (see Appendix Table A3). If DFs made effort (holding social incentives constant), being in contact with a DF can be hypothesized to correlate with higher knowledge scores of other farmers.

### 3.4 Experimental auctions

One month after the training, at the start of the rains, the DF and the 11 randomly selected co-villagers were invited to participate in an experimental auction event held in each study village. During the invitation, they were informed that they would be taking part in a market exercise involving auction of vines/seed of improved sweetpotato varieties and that they needed to bring^2^ along some money to buy vines in case they won. The auctions were designed to assess farmer demand (measured as willingness to pay (WTP)) for improved varieties, assuming that they had received information about them from the DF.

We used the Vickrey second-price experimental auction (Vickrey, 1961) to elicit farmers willingness to pay for improved sweetpotato varieties. In a Vickrey auction, participants submit sealed bids simultaneously, the highest bidder wins the auction but pays the second highest price, and there is only one winner for each auction event. This auction method has been widely used to elicit farmer preferences for various improved agricultural technologies (Lusk et al., 2004, Corrigan and Rousu, 2011). It is incentive-compatible and easy to apply in different contexts.

The auction process was organized as follows: The participants were introduced to the exercise and informed that: i) there would be four rounds of auctions, ii) they would use their own money to purchase the product if they won, iii) a binding round in which there would be a purchase will be randomly selected at the end of four rounds, and iv) only one farmer would win the auction and would be asked to pay the second highest price of the product auctioned in the randomly selected round. All the 732 sampled participants were invited to participate in the auction events.

The products auctioned were four improved varieties of sweetpotato namely, Narospot 1, Naspot 13, Ejumula and Tanzania. Each product was presented in a bundle of 200 vine cuttings of 30 cm. Only one variety was auctioned in each round. The auctioneer explained the auction process and demonstrated how it works with two bar soaps of different colors (blue and cream), including providing the cheap talk to encourage truthful (incentive-based) bidding, and how bids are recorded. Data from this demonstration/practice exercise were recorded. Once assured that participants had understood the auction process, the auctioneer introduced the first product and proceeded with the auction following the narrative provided in the auction protocol (Appendix A4). In each round, the auctioneer displayed the product, gave its description in terms of quality (e.g., disease free, yield ability, whether vitamin-A rich, pest and disease tolerance, sensory characteristics) and invited the participants to inspect the product (touch, smell, see, taste the leaves). Pictures were used to demonstrate and reinforce verbal narratives of complex and technical concepts including pest/disease symptoms, sweetpotato skin and flesh color, and root size. The auctioneer then repeated the cheap talk, and then asked each participant to record the price they would pay for the product as described in bidding sheets. The next three rounds, one for each of the remaining three products, proceeded similarly. The order in which products were presented was randomized in each auction to minimize the potential order effect (Norwood and Lusk, 2011).

Following Maredia et al. (2019), at the end of the four bidding rounds, a *binding round* was randomly selected, and bids arranged in descending order starting with highest bid. The highest^3^ bidder in that binding round purchased the product auctioned in the binding round but paid the second highest price/bid using own money. The auctions were conducted in April and May 2022. In addition to the data on bids, we also administered pre- and post-auction survey tools that collected data on various aspects of farmer, farm, and sweetpotato production and marketing (see details below).

### 3.5 Data and summary statistics

Data used in this study were collected during a survey that was split into two parts. The first part administered a pre-auction questionnaire before the auction started. It included modules about farmer and household characteristics, membership to farmer associations, social networks, preferences for sweetpotato planting materials, household income and assets, knowledge about sweetpotato farming, and risk preferences. The second part administered a post-auction questionnaire to collect data about sweetpotato farming practices, farmer perceptions of production constraints, pest and disease incidence, trust, and information about the interaction between the DFs and other farmers. In total, we interviewed 645 farmers including 59 DFs and 586 other farmers. We faced minor attrition caused by one farmer failing to show up during the training and another one not available during the auction. In both cases, the causes were not related to the experiment as both had traveled outside the village. In addition, 85 farmers representing 12.6% of the original sample of the co-villagers did not attend the auction. Using a dummy variable equal to one if attrition is present and zero if otherwise, a *test of differences in proportions* showed that attrition was not systematically different between the treatment and control group indicating that attrition was random and not related to the intervention (see Appendix Table A1). In addition to this test, the summary statistics presented in Table 1 show that characteristics of treatment and control group participants are mostly similar. Because these summary statistics are measured after attrition, we rule out that attrition is a major concern in our study.

**Table 1:** Summary statistics by treatment group.

	Whole sample	No public recognition of DF effort	DF promised public recognition	*p*-value
*Panel A: Individual and household characteristics*				
Respondent is male	0.67 (0.47)	0.72 (0.45)	0.63 (0.48)	0.019
Age of the respondent (years)	41.83 (16.31)	41.23 (16.65)	42.41 (15.95)	0.374
Education of the respondent (years)	5.85 (3.81)	5.86 (3.60)	5.84 (4.03)	0.957
Household size	7.32 (3.27)	7.25 (3.25)	7.39 (3.29)	0.574
Number of infants in the household	1.71 (1.33)	1.78 (1.38)	1.64 (1.27)	0.199
Main occupation of the respondent is farming	0.91 (0.28)	0.91 (0.29)	0.91 (0.28)	0.817
Experience in sweetpotato farming (years)	22.92 (15.90)	22.23 (16.39)	23.65 (15.36)	0.255
Membership to a farmers group	0.16 (0.37)	0.16 (0.37)	0.17 (0.37)	0.961
Distance to the nearest main market (walking minutes)	79.43 (53.58)	82.28 (54.10)	76.44 (52.95)	0.167
Distance to the nearest main road (walking minutes)	24.71 (29.27)	23.82 (27.95)	25.63 (30.61)	0.434
Access to lowlands	0.65 (0.48)	0.63 (0.48)	0.68 (0.47)	0.193
Number of sweetpotato varieties grown in last season	1.44 (1.25)	1.45 (1.29)	1.43 (1.20)	0.791
Degree of risk aversion	2.67 (1.92)	2.57 (1.92)	2.77 (1.92)	0.182
Saves planting material from own or neighbor’s farm	0.74 (0.44)	0.76 (0.43)	0.72 (0.45)	0.248
*Panel B: Experience with diseases and pests*				
Farmer has experienced sweetpotato virus disease	0.76 (0.43)	0.80 (0.40)	0.73 (0.45)	0.037
Farmer has experienced Alternaria blight	0.71 (0.46)	0.73 (0.44)	0.68 (0.47)	0.133
Farmer has observed weevils	0.87 (0.33)	0.90 (0.30)	0.85 (0.36)	0.078
Farmer has experienced millipedes	0.76 (0.43)	0.76 (0.43)	0.76 (0.43)	0.881
Farmer has experienced whiteflies	0.53 (0.50)	0.53 (0.50)	0.53 (0.50)	0.942
Farmer has experienced bacterial wilt	0.67 (0.47)	0.70 (0.46)	0.64 (0.48)	0.113
Unavailability of disease- and pest-free sweetpotato varieties	4.17 (1.10)	4.11 (1.09)	4.24 (1.11)	0.118
*Panel C: Access to information*				
Limited access to agricultural information is a constraint to sweetpotato production	3.66 (1.28)	3.55 (1.33)	3.77 (1.22)	0.037
Has received training on sweetpotato production	0.17 (0.38)	0.19 (0.40)	0.15 (0.36)	0.132
Source of information is neighbors	0.85 (0.36)	0.83 (0.38)	0.87 (0.34)	0.165
Source of information is the National Agricultural Advisory Services (NAADS)	0.12 (0.33)	0.12 (0.02)	0.12 (0.33)	0.982
Source of information is radio	0.59 (0.49)	0.61 (0.49)	0.57 (0.50)	0.293
Source of information is phone	0.15 (0.36)	0.15 (0.35)	0.16 (0.36)	0.721
Number of villages	61	31	30	
Number of observations	645	330	315	
*p*-value of joint orthogonality test				0.112

*Notes*: In parentheses are standard deviations.

Table 1 presents the summary statistics of the survey data per treatment group, including individual and household characteristics, experiences with diseases and pests, and access to information. Differences between the two groups are small in magnitude. We performed an *F*-test of joint orthogonality using a logit, which tests whether the observable characteristics in Table 1 are jointly unrelated to treatment status. We cannot reject this null hypothesis (*p*-value = 0.112), suggesting that the randomization succeeded in achieving balance for observables across the experimental arms. As suggested by Briz et al. (2017), we further conducted balance tests using data from training rounds implemented before the real auction with sweetpotato varieties. To avoid priming or anchoring participants, we used an unrelated product (blue and cream coloured bar soaps) in the training rounds. Table 2 presents summary statistics by treatments for both soaps. Formal non-parametric testing cannot reject the null hypothesis of equal distributions for the blue coloured bar soap (Two-sample Wilcoxon rank-sum Mann-Whitney test; *z* = 1.57; *p* = 0.12) and the cream coloured bar soap (Two-sample Wilcoxon rank-sum Mann-Whitney test; *z* = 0.73; *p* = 0.47), further suggesting that we can compare the treatment and control groups.

**Table 2:** Summary statistics of the training round bids by treatment.

	Median	Mean	SD	Min	Max
Blue coloured bar soap: Control	3,000	3,468	2,877	500	40,000
Blue coloured bar soap: Treatment	3,000	5,309	2,214	500	15,000
Cream coloured bar soap: Control	5,000	3,186	1,898	0	20,000
Cream coloured bar soap: Treatment	5,000	5,216	2,629	0	30,000

Most sample respondents are male. Our respondents are 42 years old and have completed six years of formal education, on average. The average household size is seven. A household had two infant children, on average. Farming was the main occupation of most of the households. Households are experienced in sweetpotato farming (22 years) and grew two varieties, on average, the previous season before the survey. About three-quarters of the households rely on saved seed from their own or neighbors’ farms. Sixty-five percent of the households have access to lowlands. Such lowlands are useful for conserving planting materials during the dry season. Farmers walk one hour and 20 min to the nearest main market and 25 min to the nearest main road, on average.

Most farmers have experienced diseases, especially the SPVD (76%) and Alternaria blight (71%). The problem of pest infestation is also prevalent: 87% of the farmers have observed sweetpotato weevils and 76% have experienced millipedes. The prevalence of pests and diseases can be caused by the reliance on seed from own and neighbors’ farms. Furthermore, on a rating scale of 1 (not a problem at all) to 5 (most serious problem), indicating the extent to which farmers perceive unavailability of disease and pest-free planting material as a constraint to their sweetpotato farming, the average score was four.

Access to information on farming is also a constraint to sweetpotato production. On a scale of 1 (not a problem at all) to 5 (most serious problem), indicating the extent to which farmers perceive limited access to agricultural information as a constraint to sweetpotato production, the average score was 3.6. Only 17% of the sample respondents had received training about sweetpotato production. Neighbors were the main source of agricultural information for 85% of the farmers. This suggests the importance of social learning in technology diffusion.

### 3.6 Data validation

Preliminary results were validated with the district and local teams. At the district level, we convened a validation workshop attended by 35 participants including frontline extension workers, county administrative staff, district agricultural production department and non-governmental organizations working on agriculture in the district. The workshop was also attended by representatives of the farmers and DFs that participated in the auctions. Preliminary findings were presented to the workshop participants to obtain feedback on the findings. Specifically, the findings relating to the effect of the intervention on knowledge and experimentation with the new varieties were discussed and opinions/views noted down. At the local level, we convened two focus group discussions in separate villages (one control, one treatment) with the farmers who participated in the study auctions, including their DFs. Each FGD was attended by 11 participants. In both FGDs, we presented key findings of the study as well as subjected opinions from the district/feedback workshop participants to interrogation by the farmers and DFs. This included opinions on what type of incentive would stimulate greater effort in sharing knowledge and experimentation with the improved varieties by DFs and co-villagers.

### 3.7 Empirical estimation

We examine the effect of public recognition plus goal setting on the main outcomes of interest, using the following equation:

(2) $$y_{\mathrm{ivc}}=\alpha +\beta {\mathrm{Treat}}_{\mathrm{ivc}}+\xi_{c}+\varepsilon_{\mathrm{ivc}}$$

where $y_{\mathrm{ivc}}$ represents the outcome of interest for farmer i in village v and county c: measures of knowledge, diffusion effort, and willingness to pay. To gauge knowledge levels, we administered a simple test focusing on the content of the training that DFs had received. Such exams are an effective approach of assessing knowledge retention by subjects (Kondylis et al., 2015). To measure diffusion effort by DFs we use a binary outcome capturing whether the DF had contacted any of the sampled co-villagers (based on survey data provided by co-villagers, not the DFs). In addition, we include an effort variable measuring the number of co-villagers with whom the DF communicated about the improved sweetpotato varieties including the farming practices learnt during the training. To minimize the tendency that co-villagers mention the DFs as contacts even when there might not have been communication, we probed for the content of their discussion. As described in Section 3.4 above, willingness to pay was measured using the bids submitted in the Vickrey second-price experimental auction. The variable ${\mathrm{Treat}}_{\mathrm{ivc}}$ denotes the treatment dummy (public recognition plus goal setting), with the training-only group as comparison group. $\xi_{c}$ captures county fixed effects. We use OLS to explain variation in knowledge, number of farmers contacted by the DFs, the likelihood of a DF contacting other farmers, and willingness to pay (by DFs and other farmers). Throughout we cluster standard errors at the village level. The coefficient β in Eq. (2) measures the causal effect of the public recognition plus goal setting on knowledge scores, effort, and willingness to pay under the identifying assumption that ${\mathrm{Treat}}_{\mathrm{ivc}}$ is orthogonal to $\varepsilon_{\mathrm{ivc}}$.

## 4 Results

### 4.1 Incentives and disseminating farmers’ knowledge and effort

Table 3 presents results of OLS regressions assessing the effect of goal setting combined with public recognition on DFs’ knowledge (column 1) and their diffusion effort (columns 2–3). We find that asking DFs to set goals and offering them public recognition has no effect on the knowledge retained by DFs three weeks after the training.^4^ All DFs received the same training. Furthermore, incentives were only communicated to the treatment group at the end of the training. It is possible, therefore, that incentives did not influence the attention paid during the training which would have otherwise influenced the amount of knowledge retained. This was done to assure that all farmers could receive the same training (to avoid strong confounds of training sessions with the treatment) without the risk of revealing the treatment.

**Table 3:** Effect of incentives on disseminating farmers’ knowledge and diffusion effort.

Incentive type	Knowledge	Effort	
		Likelihood of DF sharing knowledge with other farmers	Number of farmers contacted by DF
	(1)	(2)	(3)
Training plus public recognition	−0.30 (0.54)	−0.19^**^ (0.07)	−1.90^***^ (0.71)
County of residence is Toroma	0.66 (0.61)	0.03 (0.08)	0.13 (0.80)
County of residence is Usuk	0.90 (0.82)	−0.09 (0.10)	−1.79* (0.99)
Constant	6.91	0.49^***^ (0.06)	4.86^***^ (0.62)
R-squared	0.04	0.16	0.14
Observations	59	586	59
Mean of dependent variable for non-incentivized DFs	7.26 [2.13]	0.47 [0.50]	4.62 [2.82]

*Notes*: OLS regression estimates. Robust standard errors clustered at village level are in parentheses. Square parentheses are the standard deviations of the control group means. ***=p < 0.01, **=p < 0.05, *=p < 0.1.

Goal setting combined with public recognition reduced the likelihood of a DF sharing knowledge with other farmers (Column 2). On average, the probability of contacting other farmers to share information decreased by 19 percentage points for the goal setting plus public recognition reward relative to the mean (0.47) for the control group. Similarly, goal setting combined with public recognition decreased the number of farmers contacted by the DF by 1.9 compared to the comparison group (mean = 4.6).

### 4.2 Incentives and other farmers’ knowledge and willingness to pay

Results of the effect of goal setting combined with public recognition on the knowledge of other farmers and their willingness to pay for the improved varieties are presented in Table 4. Goal setting combined with public recognition had no effect on the knowledge scores of other farmers (Column 1). The 0.35 points decrease in knowledge scores of other farmers, corresponding to 8.6% reduction (given that the mean knowledge score in the control group was 4.07), in the goal setting combined with public recognition treatment group relative to the control group is not statistically significant. Further, we find that goal setting combined with public recognition incentive reduced other farmers' willingness to pay for two out of the four varieties. Willingness to pay for Ejumula decreased by UGX 749 in the goal setting combined with public recognition treatment relative to the mean (UGX 3,996) for the respondents in the training-only villages (Column 2). This corresponds to 18.7% decrease. Similarly, willingness to pay for Naspot 13 decreased by UGX 797 in the goal setting combined with public recognition treatment, corresponding to 16.6% reduction, relative to the mean (UGX 4,794) for the respondents in the training-only villages (Column 5). This effect is only statistically significant at the 10% level. The effect of goal setting combined with public recognition on other farmers’ willingness to pay for Tanzania and Narospot 1 was null (Columns 3–4). The point estimates in the goal setting combined with public recognition treatment are negative and correspond to UGX −346 for Tanzania and UGX −318 for Narospot 1.

**Table 4:** Effect of incentives on other farmers' knowledge and willingness to pay for improved sweetpotato varieties.

Incentive type	Other farmers’ knowledge	Willingness to pay			
		Ejumula	Tanzania	Narospot1	Naspot13
	(1)	(2)	(3)	(4)	(5)
Training plus public recognition	−0.35 (0.26)	−748.52^**^ (297.02)	−346.36 (350.46)	−317.68 (371.91)	−796.87* (415.48)
County of residence is Toroma	0.07 (0.32)	671.24^**^ (333.07)	360.32 (456.07)	340.01 (401.79)	284.67 (416.03)
County of residence is Usuk	−0.35 (0.28)	−393.33 (502.49)	−134.13 (605.60)	−721.81 (548.93)	−313.15 (760.81)
Constant	4.14 (0.20)	3,963.46^***^ (230.69)	3,977.82^***^ (290.98)	4,090.36^***^ (319.28)	4,814.81^***^ (357.47)
R-squared	0.01	0.03	0.00	0.01	0.01
Observations	586	586	586	586	586
Mean of dependent variable for other farmers in villages where DFs were not incentivized	4.07 [1.71]	3,995.67 [2,872.95]	4,013.33 [3,249.35]	3,983.00 [3,285.55]	4,793.83 [4,249.33]

*Notes*: OLS regression estimates. Standard errors clustered at village level are in parentheses. Square parentheses are the standard deviations of the control group means. ***=p < 0.01, **=p < 0.05, *=p < 0.1.

### 4.3 Robustness check

We perform several robustness checks to probe our results further. First, we conduct regression analysis to assess the effect of goal setting combined with public recognition on DF’s willingness to pay. Results in Table 5 show that goal setting plus public recognition did not affect DFs’ willingness to pay for any of the four improved varieties of sweetpotato. Disseminating farmers that were asked to set goals and were offered public recognition had the same willingness to pay as their counterparts in the control group.

**Table 5:** Effect of incentives on disseminating farmers’ willingness to pay for improved sweetpotato varieties.

Incentive type	Ejumula	Tanzania	Narospot1	Naspot13
	(1)	(2)	(3)	(4)
Training plus public recognition	−1,110.26 (849.53)	68.12 (814.31)	193.51 (1,155.40)	12.45 (1,347.40)
County of residence is Toroma	33.80 (931.06)	−574.11 (873.00)	−1,327.50 (1,233.25)	140.03 (1,439.24)
County of residence is Usuk	−3,448.04^***^ (865.23)	−1,524.25 (1,014.38)	−3,074.20^***^ (1,068.64)	−2,468.34* (1,407.84)
Constant	6,831.11^***^ (807.19)	5,553.81^***^ (674.99)	6,116.15^***^ (1,006.27)	7,114.61^***^ (1,264.84)
R-squared	0.16	0.03	0.08	0.04
Observations	59	59	59	59
Mean of dependent variable for non-incentivized DFs	6,033.33 [3,759.89]	5,083.33 [3,186.94]	5,133.33 [4,368.65]	6,566.67 [5,165.76]

*Notes*: OLS regression estimates. Robust standard errors clustered at village level are in parentheses. Square parentheses are the standard deviations of the control group means. ***=p < 0.01.

Second, we perform a placebo test by regressing training round bids on the treatment dummy. If the coefficients on the treatment dummy are significantly positive or negative, it would indicate the presence of unobserved heterogeneity, which could introduce bias. Results in Table 6 indicate no statistically effect of goal setting and public recognition on training round bids, suggesting that our estimates are not affected by such bias.

**Table 6:** Placebo test: Effect of incentives on training round bids.

Variable	Blue coloured bar soap	Cream coloured bar soap
Training plus public recognition (PR)	−274.54 (191.37)	−66.33 (221.30)
Cluster size	61.30 (81.80)	227.67^**^ (113.67)
Constant	2,808.14^***^ (896.87)	2,859.90^**^ (1,240.61)
R-squared	0.004	0.01
Observations	645	645

*Notes*: OLS regression estimates. Standard errors clustered at village level are in parentheses. ***=p < 0.01, **p < 0.05.

Third, we statistically consider selection on observables to understand the risk of omitted variable bias. We adopt the methodology developed by Oster (2017), which is the extension of the idea of Altonji et al. (2005). According to Oster (2017), if the assumption of proportional selection holds (i.e., that the relationship between the outcome and the observed control variables informs the relationship between outcome and unobservables), then changes in the magnitudes of the coefficients and value of $R^{2}$ can tell us about the size of omitted variable bias. There are two ways to conduct this test. The first approach is to calculate the value of δ (in Eq. (3) below), which is the degree of selection on the unobservables relative to the observables that would be needed to drive our estimated coefficients to zero. δ is mathematically defined as:

(3) $$\delta \approx \frac{\left(\tilde{\beta }-\beta^{*}\right)\left(\tilde{R}-R^{o}\right)}{\left(\beta^{o}-\tilde{\beta }\right)\left(R_{\max}-\tilde{R}\right)}$$

where $\beta^{o}$ is the coefficient of the treatment dummy and $R^{o}$ is the $R^{2}$ value in the simple regression of outcome on treatment; $\tilde{\beta }$ and $\tilde{R}$ correspond to those in the regression with all observable controls included. $\beta^{*}$ is the targeted value of the coefficient (e.g., zero). $R_{\max}$ corresponds to $R^{2}$ in a hypothetical regression containing all observable and unobservable controls. Oster (2017) recommends using 1.3 $\tilde{R}$ as the value for $R_{\max}$; a δ value of 1 is considered an appropriate cutoff. The second approach to conducting this robustness test is to estimate coefficient bounds. One bound is $\tilde{\beta }$, the value of β when δ=0. The other bound is $\beta^{*}$, the value of β when δ=1 and ${R^{2}=R}_{\max}$. If the estimated coefficient bounds interval does not include zero, the estimates are robust to unobservables. We use both approaches to test for the robustness of our estimates.

Table 7 shows the estimates of robustness to unobserved heterogeneity as per the procedure of Oster (2017). Overall, both the value of δ and the coefficient bounds point to robustness in our estimates. In our case, δ<0 for all the outcomes because of the negative sign of the estimated coefficients across the outcomes. In absolute terms, the values are greater than 1, indicating that the effects can be considered robust to unobserved heterogeneity. Similarly, the coefficient bounds intervals do not contain zero, which also implies that the estimates are robust.

**Table 7:** Oster bounds and selection on observables.

Outcome variable	δ for β=0	Coefficient bounds	
		No control	With controls
Knowledge of other farmers	−4.43	−0.29 [0.007]	−0.35 [0.015]
Willingness to pay for Ejumula	−1.92	−537.28 [0.010]	−748.52 [0.030]
Willingness to pay for Tanzania	−1.92	−240.96 [0.001]	−346.36 [0.005]
Willingness to pay for Narospot1	−3.67	−156.43 [0.001]	−317.68 [0.013]
Willingness to pay for Naspot13	−1.16	−692.09 [0.009]	−796.87 [0.012]

*Notes*: The control variables in the regressions include county fixed effects. In square parentheses are the R-squared (R^2^) values.

## 5 Discussion

Diffusion of agricultural innovations is crucial for sustainably transforming agri-food systems in Sub-Saharan Africa and improving livelihoods. The diffusion of innovations with potential welfare-enhancing benefits is not automatic. Incentives play a crucial role in motivating agents to expend costly effort to reach out to others with information about innovations (Ashraf et al., 2014, BenYishay and Mobarak, 2019, Shikuku et al., 2019). Our finding that goal setting combined with providing public recognition as an incentive reduced the effort made by DFs to disseminate agricultural information contradicts these studies. Instead, the results suggest possible crowding out effects consistent with Savary and Goldsmith, 2020, Wu and Jin, 2020, and Gneezy et al. (2011). Consistent with our theoretical framework, several reasons can explain our findings. First, if agents expect larger material rewards, failure to meet these expectations can backfire (Gneezy and Rustichini, 2000). We conducted follow up qualitative interviews to probe this possibility. Participants in the focus group discussions (FGDs) confirmed that public recognition of effort is perceived as an attractive incentive. We therefore rule out that public recognition might not be “good” enough for the DFs.

The second possible explanation of our results is that other farmers might perceive DFs’ action as a type of self-benefit (Barasch et al., 2016). If “jealous” co-villagers thought that the DFs would “use” them to gain popularity and attract benefits to themselves in the future, they may not pay attention to the DF. A few participants in the qualitative FGDs reported this as the reason for low effort. However, all the DFs were already “famous” and recognized by the communities as champion farmers and role models. Contrary to the “jealousy” argument, it is possible that although public recognition is appreciated as a reward for effort, it’s incremental effect on reputation may not be much. Several studies have indeed shown that the selection criteria for DFs matters (e.g., BenYishay and Mobarak, 2019, Shikuku and Melesse, 2020). Related to this and as a third reason, providing public recognition to already popular DFs can reduce effort because of over-justification effect. In this context, over-justification can take two main forms. The first form is DFs questioning the motive for incentivizing them, especially if the benefits associated with the innovation have already been explained to them. The second form is whereby DFs protest against public overemphasis of their role as publicly recognized champion farmers when they are already well-known by the community.

The finding that public recognition did not influence experimentation by DFs has important implications. In our context, experimentation is proxied by farmers’ willingness to pay for the varieties. Models of social learning are premised on the assumption that neighbors learn and adopt an innovation after observing the behavior of the DF (e.g., Acemoglu et al., 2008). Our findings raise the question as to whether adoption can happen simply because DFs disseminate information about an innovation even if they themselves did not try out the innovation. We also observe weak effects of public recognition combined with goal setting on experimentation by other farmers. Shikuku et al. (2019) found similar results and argued that DFs may require a considerable period of time to decide on implementation of an innovation, especially if it is not immediately obvious that the innovation would be welfare-enhancing to all co-villagers. Most recently, Balew et al. (2022) also document null effects of incentives on experimentation.

One possible limitation of our research is the lack of incentive compatibility of the 2nd price auction. DFs may communicate to farmers the expected yields and incomes from the improved varieties, therewith inducing correlated common values and hence limiting the individual valuations (see Krishna, 2009 for a discussion). We can only speculate where on a spectrum from individual values to interdependent common values our participants are positioned. However, as DFs mostly communicate benefits in agronomic terms (rather than specific yields), and as farmers are used to process information received from DFs and relate it to their own farms. In addition, the DFs had grown and not harvested the improved varieties, at the time of auctions, hence knowledge about higher yielding abilities of these varieties was limited to the information learned during the training. Therefore, the loss in incentive compatibility, if any, is likely to be small.

## 6 Summary and conclusions

Millions of households in developing countries rely on agriculture for a living. Improving the livelihood of these households will require transition from low-input subsistence farmers to use of yield-increasing agricultural technologies. The use of improved seed varieties was credited with large yield improvements during the green revolution. However, in developing countries, the majority of farmers continue to rely on recycled seed from their own or local sources. Such seeds tend to be heavily infected with pests and diseases, resulting in large yield gaps. The use of improved varieties in developing countries is often impeded by the lack of access to such seed and information regarding their superior performance. In this study, we used a field experiment to examine the effect of public social recognition combined with goal setting on the diffusion of agricultural knowledge and smallholder farmers’ uptake of quality certified seeds. Recent literature has also indicated that providing incentives to communicators can spur information sharing and uptake of agricultural technologies. We focused on sweetpotato, one of the root and tuber crops, where only 6% of farmers use improved varieties. We relaxed the seed access and information/knowledge constraint by providing improved varieties in the study villages and training to carefully selected champion/disseminating farmers (DFs) who were then linked to co-villagers. Half of the DFs, the treatment group, received a social incentive in form public recognition and also set goals regarding how to reach co-villagers. We find that this social incentive combined with goal setting had no effect on knowledge and also experimentation by DFs. We also find that the treatment had no effect on willingness to pay for improved seed, our proxy for experimentation, by co-villagers. These findings are contrary to recent literature on social learning and technology uptake that has tended to focus mainly on material incentives. Rather than induce effort, the combination of goal setting and public recognition acted to crowd-out effort, in line with other studies. We therefore conclude that a social incentive combined with goal setting by established progressive farmers already enjoying a certain degree of public recognition is not sufficient to induce effort in disseminating knowledge and experimentation with improved agricultural technologies.

The implication for policies and efforts promoting improved agricultural innovation with food security and nutrition benefits is that identifying optimal ways to incentivize DFs is important but not straightforward. While nudges are increasingly used to influence adoption of appropriate behavior, nudges in the form of goal setting combined with social incentives can backfire when the selected DFs are already popular. Importantly, this crowding-out effect can cause less than optimal use of agricultural innovations, consequently compromising efforts to increase food security and reduce malnutrition.

## CRediT authorship contribution statement

**Julius Okello:** Conceptualization, Funding acquisition, Investigation, Methodology, Project administration, Resources, Supervision, Validation, Writing – original draft, Writing – review & editing. **Kelvin Mashisia Shikuku:** Conceptualization, Formal analysis, Investigation, Methodology, Project administration, Validation, Writing – original draft, Writing – review & editing. **Carl Johan Lagerkvist:** Conceptualization, Funding acquisition, Methodology, Resources, Writing – review & editing. **Jens Rommel:** Conceptualization, Formal analysis, Methodology, Writing – original draft, Writing – review & editing. **Wellington Jogo:** Conceptualization, Methodology, Writing – review & editing. **Sylvester Ojwang:** Data curation, Formal analysis, Investigation, Project administration, Writing – original draft, Writing – review & editing. **Sam Namanda:** Conceptualization, Methodology, Project administration, Validation, Writing – review & editing. **James Elungat:** Methodology, Project administration, Resources, Validation, Writing – review & editing.

## Declaration of Competing Interest

The authors declare that they have no known competing financial interests or personal relationships that could have appeared to influence the work reported in this paper.

## Figures and Tables

**Table A1:** Formal test for attrition bias.

Variable	Training plus public recognition	Control	*p-*value
Attrition is present (1 = yes; 0 = otherwise)	0.13 (0.02) [0.33]	0.11 (0.02) [0.32]	0.613
Observations	360	372	

*Notes*: Results of *a test of differences in proportions*. Standard errors are reported in parentheses. In square parentheses are standard deviations.

**Table A2:** Effect of being contacted by a disseminating farmer on willingness to pay for improved sweetpotato varieties.

Incentive type	Ejumula	Tanzania	Narospot1	Naspot13
	(1)	(2)	(3)	(4)
Farmer was contacted by a trained DF	472.69* (276.21)	307.50 (321.99)	205.34 (288.32)	509.39 (411.82)
County of residence is Toroma	474.66 (345.04)	271.63 (473.38)	256.70 (410.93)	75.56 (429.06)
County of residence is Usuk	−306.96 (527.45)	−87.07 (627.69)	−684.78 (559.86)	−220.70 (797.24)
Constant	3,460.02^***^ (250.16)	3,708.45^***^ (255.34)	3,874.76^***^ (292.94)	4,276.32^***^ (365.99)
R-squared	0.02	0.00	0.01	0.01
Observations	586	586	586	586

*Notes*: OLS regression estimates. Robust standard errors clustered at village level are in parentheses. ***=p < 0.01, *=p < 0.1.

**Table A3:** Effect of being contacted by a disseminating farmer on knowledge.

Incentive type	Knowledge scores
	(1)
Farmer was contacted by a trained DF	0.83^***^ (0.22)
County of residence is Toroma	−0.00 (0.25)
County of residence is Usuk	−0.26 (0.24)
Constant	3.66^***^ (0.14)
R-squared	0.06
Observations	586

*Notes*: OLS regression estimates. Robust standard errors clustered at village level are in parentheses. ***=p < 0.01.

## Appendix

See Table A1, Table A2, Table A3.

## Appendix 4 Auction script

Researcher: Good morning/afternoon; Welcome to this market exercise, we thank you for your participation. My name is________and with me today is my colleagues__________.

We are carrying out joint research led by International Potato Center and Katakwi District Agricultural Office to understand farmers acquisition of sweetpotato planting material and the varieties they they like. We are therefore part of a research team, not sales persons for any company; but as part of this market exercise we may sell you some sweetpotato seed. Today we are going to carry out a series of auctions of the different types of sweetpotato vines. We will ask you to make purchase decisions just as the one that you would in the market if you were to purchase vines from the market or neighbor or other sweetpotato farmers. During the course of the market exercise you will have an opportunity to buy the vines we will be auctioning, if your bid is the highest among other bidders bid. But remember to be truthful and honest about your decisions.

As the first part of this activity, you will complete a pre-auction survey administered by one of our team members.

From this point onwards we will issue you with piece of paper with a number on it. That number will be your ID No. Throughout the market exercise, we will identify you by the ID number on your paper; therefore, do not write your name anywhere in the answer/bid sheets we will give you for recording/writing your responses. Your responses will therefore only be known to us and will be confidential. We will give you more details on this shortly.

The whole market exercise, including the auction sessions, will last **approximately 1 h and 30 min**.

## [PAUSE]

Do you have any questions?

## [Wait, and address any questions raised before proceeding]

Now we will proceed to start the market exercise by asking you individually a few questions. This individual interview session will last about 15 min. We will inform you when it is your turn to be interviewed. If you are already interviewed, please remain sitted. Thank you.

## [Assemble all the auction participants into the auction venue/hall.]

Researcher: Thank you all, again, for agreeing to participate in this study. We will now embark on a series of group auctions. At this time, we would like you to confirm that i) you have completed the first one-to-one (pre-auction survey) interview and ii) that you can dedicate at least 1 h for the group auction workshop. It would be unfortunate if someone leaves before finishing.

## [Give time for people to adjust. Before starting …..

• *Ask if there are any participants who have not completed the pre-auction survey.*
• *If someone cannot participate the whole time period, please request that they leave. Listeners are not allowed.*
• *Make any final reordering of the group*.]

Researcher: We are going to begin the auctions now. At this time, we request that you please turn your cell phones into silent mode or off. [*Allow time to turn cell phones off*].

Today you will participate in an auction activity divided into 4 rounds. In each round, you will have a chance state your maximum bid/price for the seed types being auctioned. At the end of the ROUND 4 (i.e., all the bidding rounds), one round will be randomly selected, and the persons with the highest bid in that round will be the buyer of the seed type that was auctioned in that round. In case of a tie in the highest bid, we will decide the buyer by tossing a coin. To guarantee the confidentiality, it is very important that you do not speak among each other during the auctions, and that you strictly follow the instructions we will be giving you.

If you have any doubts/questions, please raise your hand and one of us will come and help you; you can ask any of us questions. It is very important that you follow the instructions; if not, you will be disqualified from further participation.

Researcher [*Say to the whole group*]: The result of this market exercise is completely confidential. To guarantee this, use only your ID number during this auction. The ID number will also serve as part of your identification when collecting sweetpotato vines today, if you win during the auction.

Because this is the time for planting sweetpotato, any vines you buy during this auction will be made available to you today and here so you can proceed and plant it.

## [Show a piece of paper with ID number for illustration purposes]

Researcher: Do you have any questions about what we have talked about so far? [*Wait and answer questions raised*].

Now, we will demonstrate to you how the auction process will work. Please pay very close attention. Don’t be afraid to ask if anything is not clear to you.

## [Practice auction]

For this practice auction, each of you will bid on the two types of bar soaps [*hold the bar soaps up*]. Like some auctions you may have participated in in the past, in this auction only one person will buy ONE bar soap which will be RANDOMLY SELECTED.

Let me walk through how you will bid and how we will determine who buy a bar of this soap.

First, we will hand out a bidding sheet like this one.

## [Hold up bidding sheet.]

On these bidding sheets you will:

i) write down your ID number.

ii) write down the maximum amount of money you would be willing to pay for each bar of soap. If you need help with writing, please raise your hand and one of us will come to help you. However, you must determine the maximum amount of money you would be willing to pay YOURSELF, without anybody’s influence.

iii) if you don’t want to bid, you’re allowed to write zero, but you will not be able to buy a bar soap even if you like/want it.

Once everyone has written the MAXIMUM amount of money they are willing to pay for each bar of soap, we will collect the bidding sheets. Then we will randomly select one of the bar soaps and move on to determine the buyer of that bar soap.

To determine who buys, we write on this flip chart [*show the flip chart*] the bids for the randomly selected bar soap from the highest to the lowest. As in some of the auctions you fttknow or have participated in, the participant with the HIGHEST bid will be the buyer. However, instead of paying his/her bid (the highest), he/she will pay the **second highest bid**. So, there will only be ONE BUYER of the randomly selected bar soap. If there is a tie in the highest bid for the bar soap, we will decide who the buyer is by tossing a coin.

So, for example, suppose that [*name an enumerator1 in the room*] bids 20, I bid 16 and [*name an enumerator2 in the room*] bids 25 for the randomly selected bar soap. We will first arrange the bids from the highest to the lowest as below:

## RANDOMLY SELECTED BAR SOAP

1. 6,000
2. 5,000
3. 3,000

In this case [*name of enumerator2* ] has the highest bid for randomly selected bar soap A and therefore gets to buy the bar soap, but he/she pays 5,000 UGX for it, not the 6,000 UGX bid.

Are there any questions?

If there are no questions, I will now ask you a few questions about what we are to do based on what I have just said. I would like to be sure you understand the process well [*Give some quiz on the auction procedure*].

Before we hand out the practice round bidding sheets, let me explain the best strategy in this type of auction. The BEST thing to do is to bid the MAXIMUM amount you are willing to pay for the product. This is because it is very likely you will actually pay LESS if you buy.

However, bidding less than what you would be willing to pay might mean that you miss out on buying the soaps at a price lower than you would be willing to pay.

Similarly, bidding more than what you would be willing to pay for the bar soaps might mean that you end up having to pay more for the soap than you really want to. For example, if you are willing to pay a maximum of 3,000 UGX, but you bid 5,000 UGX and the second highest bid ends up being 4,000 UGX, then you would pay 4000 UGX – more than you were willing to!

Overall, your best strategy is to bid the MAXIMUM amount you are willing to pay for the product and you feel reflects real value of that product to you.

## [Ask if this is clear, and wait.]

We will now hand out the bidding sheet to each of you.

## [Hand out bidding sheet to each participant]

First, go ahead and write down your ID number from the card you received at the start– this helps us keep track of everyone’s bids- and your bid for a bar of soap. Please do not talk with others until we have collected the bids. If you do, you will be disqualified. Instead ask any one of us if you have a question.

## [Collect bidding sheets, making sure that bids and numbers are entered and legible and that the bid is in whole UGX increments (i.e., 5,000.50 UGX is not a valid bid).]

Now we will display all the bids on the flip chart from the maximum to minimum.

## [Write bids on a flip chart in a descending order]

The highest bid/price is UGX___________________.

This buyer pays the 2nd highest price which is UGX________________________.

Therefore, this bar of soap was won/bought by [*name ID of the winner*].

[*Ask*] Are there any final questions on how this auction works?

[*Give time to address all questions about the illustration of the auction procedure. Ask again if there are still further questions on the procedure*] [*Based on your judgement, a second illustration can be given, but this will require use of a different auction item – not bar soap*].

Any final questions?

## [Seed auction begins]

Now, you understand how the auction will operate. The vine auction will be very similar to the practice auction we just did with the bar soaps, but please note the following:

First, you will be bidding to purchase [1 bundle] of sweet potato vines each containing 100 pieces of 30-cm-long cuttings. The bundles are for FOUR different seed/vine types (or products). We will have 4 rounds of auctions. You will therefore be making a total of 4 bids – one for each seed type/product.

Second, at the end of the 4 rounds, one round will be will be randomly selected and the bidders with the highest bids for the product in that round will be the buyer of that product.. They will however pay the second highest price for that product.

Third, as in the practice auction we have illustrated, you will pay for the vine type (product), if you bid highest for it, using your own money.

Fourth, buyers will be issued with the vines of the seed type they have bid highest today and here (at the end of the auction sessions).

Are there any questions? [*Wait and answer any questions raised*] [*If no questions, give brief quiz on the actual auction procedure, then continue*].

[Auction round 1 begins].

Now, let’s go to the first round. Before we hand out the bidding sheets for this round, let me just remind you that your best strategy is to bid the MAXIMUM amount you are willing to pay for each seed type (product). [*Repeat the cheap talk here*].

Also, remember that since you will only be able to buy ONE randomly selected seed type, you do NOT need to try and spread your money across the 5 seed types, thus place lower bids than you would really wish to.

As before, please do not talk with others until we have collected the bidding sheets. Otherwise, you will be disqualified from further participation in the auction.

## [Hand out bidding sheet. Ask if anybody has not received the bidding sheet before you continue. If none, ask…..]

Are there any final questions? [*Wait and respond to any questions before continuing.*].

Now go ahead and write down your ID number (from the card) on your bidding sheet.

[*Give the description of the FIRST product*]: In this round, you will bid for the first product (vine type) [… *state name*…] before us. This seed type is … [*give a product description as in the study design section above*]….. Take a few minutes to look closely at this seed type [*name*] to assess it, as you would do when selecting vines to plant to plant in your garden, before you bid. You can feel, smell, and touch but DO NOT discuss it with others. Based on your OWN assessment of the this seed type, write down in your bidding sheet the maximum bid you are willing to pay for each seed type. Remember that bidding is in multiples of 1000 UGX.

## [Allow time for participants to assess the first product and write their bid on the bid form.]

### [Collect bidding sheets, making sure that bids and numbers entered are legible and in multiples of 1,000 UGX]

Thank you very much for writing down your bids.

Bid price for Seed type #1 [*name*]is [UGX]__________________.

Thank you very much, now let’s go to the second auction.

[Auction round 2 begins].

We will now move to the second seed type (product) in this second round. Please listen carefully to me.

**Product #2** [*Hold up the bundle*] is vines of seed type 2 [state name] obtained from a ….. [*Give product #2 description as in the design section above*].

Now take time to think about this seed type, its advantages (i.e., what it offers)and what that means to you as a farmer. Feel free, again, to assess/examine the product/seed type (look closely, feel, touch, smell) as you would typically do when obtaining vines to plant. I remind you that discussing the product with another participant is NOT allowed.

## [Allow time for the participants to assess and evaluate the products in view of the new information.]

Now, write down in your bidding sheet the MAXIMUM amount you are willing to pay for it, given the information you have just received.

Bid price for product #2 is__________________________.

[Auction Round 3 begins].

In this round, we now present the 3rd seed type. But before we do that, I want to give you some information about it.

**Product #3** [*hold it up*] [*Give the description of this seed type as in the study design above*].

With this information in mind, I would like you to first examine/assess this seed type, as you would do when obtaining sweetpotato vines to plant. Once you have done so, and are satisfied, you will be asked to write down, on bidding sheet, the MAXIMUM amount of money you are willing to pay for it.

Let me again remind you that your best strategy is to bid the MAXIMUM amount you are willing to pay for this seed type. [*Repeat the cheap talk*.].

## [Allow time for respondents to reflect on this information]

Now, use the bidding sheets you have to write down your bids. Let me remind you that you are not allowed to discuss with your fellow participants what you plan/want to do.

## [Allow time for participants to write their bids.]

Bid price for product #3 is__________________________.

Thank you all very much!

## REPEAT THE EXERCISE FOR ROUND 4

[Auction Round 5 begins].

During this FINAL round, we will bid for the last vine/seed type [*name*] (products) which is here before us.

**Product #5** [*Hold up the bundle*] is [*Give product information as in the study design section above*].

Now, use the bidding sheets you have to write down your bid. Let me remind you, again, that your best strategy is to bid the MAXIMUM amount you are willing to pay for this seed type [*Repeat the cheap talk*].

## [Allow time for participants to write their bids. When everybody is done, collect the bidding sheets]

Bid price for Product #5 is UGX__________________.

## Selection of binding round/product

Now we will proceed to select the round that allows the one with the highest bids to purchase the vines. This round will be selected randomly. We will start by giving the auction rounds numbers so that Round #1 = 1,… Round #5 = 5. Then one of you will pick the binding as you normally do in a lottery.

## [Have one participant randomly pick a number from folded papers with 1, 2, 3, 4. 5 representing the rounds. Show the number selected.]

The selected Round is__________________.

The product/seed type we auctioned in this selected Round was [*specify as appropriate*]:

Variety name:_____________________.

Now, we will arrange all the bids in this round for this variety/product.

## [Arrange the bids for the product/seed tye in a descending order on a flip chart/board. Announce the highest bid and the price to be paid]

Remember that ONLY the highest bidder production this round gets to buy the product, but she/he pays the 2nd highest bid price. Remember also that the highest bidder buys (or pays for) the product using own money. Looking at the board/flip chart [*if there is a tie in highest bid, decide buyer by tossing a coin*]:

The highest bid price of seed type [*name*] in this winning round is [UGX]_____________.

The 2nd highest bid price for this product [*name*]is_________________.

## [Announce the bidder with the highest bid for each product. Then collect the payments and hand the purchased vines]

END OF THE AUCTIONS

Thank you all very much! We have finished with auctions. You have done a terrific job!

We will now take a break. During this break, some refreshments will be served. It will be followed by PART 3 of the study during which we will ask each of you individually, again, some few additional questions about yourself and household and your sweetpotato farming practices. So please don’t leave. Also, if you didn’t do the first interview, you should do it at during that session.

After the individual (pre and post) interviews you will receive a token of appreciation for coming to the auction workshop to help you meet your transportation cost to this workshop. We will also give each of you a document/handout that will help you understand how to be a better sweetpotato farmer.

[Part 3 begins].

Each participant is interviewed using the post-auction questionnaire. The participant is then given transport compensation, handout on basic sweetpotato agronomics practices, and allowed to leave at his/her own pleasure.

## Footnotes

1 We recognize that providing training is an incentive in itself. However, because DFs in both T0 and T1 receive the same training, exogenous variation only comes from the allocation of only DFs in T1 to set goals and receive social recognition as an incentive for effort provision.

2 That is, they would have to spend their own money to purchase vines if they won.

3 In case of a tie in the highest bid, the winner would be decided by tossing a coin.

4 Note that we performed expedited ex-ante power calculations for the experiment, working with the strongly simplified assumption of a metric normally distributed outcome and uncorrelated errors (i.e., an unpaired *t*-test). Under these assumptions, sample sizes of 315 and 330 allow us to detect effect sizes as small as d = 0.23 for α = 5% and P = 0.8 or a two-sided test. Given the differently structured data, we should be aware, however, that the minimum detectable effect size might be larger.
